# Whole-genome sequencing data of *Corynebacterium diphtheriae* isolated from diphtheria outbreaks in Indonesia

**DOI:** 10.1016/j.dib.2022.108460

**Published:** 2022-07-14

**Authors:** Vivi Setiawaty, Nelly Puspandari, Ratih Dian Saraswati, Dwi Febriyana, Tati Febrianti, Yuni Rukminiati, Fauzul Muna, Fitriana Fitriana, Dodi Safari, Rahadian Pratama, Lisa Andriani Lienggonegoro, Sunarno Sunarno

**Affiliations:** a National Referal Infectious Diseases Hospital Prof. Dr. Sulianti Saroso, Jakarta, Indonesia; b Centre for Research and Development of Biomedical and Basic Health Technology, National Institute of Health Research and Development (NIHRD), Ministry of Health, Jakarta, Indonesia; c National Research and Innovation Agency (BRIN), Jakarta, Indonesia; d Department of Biochemistry, Faculty of Mathematics and Natural Sciences, Bogor Agricultural University (IPB University), Bogor, Indonesia; e Centre for Health Reselience and Resource Policy, Health Policy Agency, Jakarta, Indonesia

**Keywords:** *Corynebacterium diphtheriae*, Whole-genome sequencing, Diphtheria outbreak, Indonesia

## Abstract

*Corynebacterium diphtheriae* (*C. diphtheriae*) is the causative agent of diphtheria. The main virulence factor of *C. diphtheriae* is diphtheria toxin, which is encoded by the *tox* gene and regulated by the *dtxR* gene. The *tox* and *dtxR* genes are used as genetic markers to identify bacteria causing diphtheria by PCR. Here, we present the whole-genome sequencing (WGS) data of 18 *C. diphtheriae* isolates from diphtheria outbreaks in different regions in Indonesia. We used these data to identify *single nucleotide polymorphisms* (SNPs) associated with the *tox* and *dtxR* genes to verify the accuracy of the PCR assay and performed molecular typing with a multilocus sequence typing (MLST) approach. The data can be used for further analyses, such as antimicrobial resistance and bacterial virulence factors.

## Specifications Table

**Table:** 

Subject	Biological Sciences
Specific subject area	Genomics
Type of data	Genome sequences data (DNA-seq raw reads) and table
How data were acquired	Illumina MiSeq sequencing platform (Illumina, San Diego, USA)
Data format	Raw sequences (FASTQ) and isolates data
Description of data collection	DNA extraction was performed using the QIAamp DNA Mini Kit (Qiagen, Hilden, Germany). DNA was quantified by Qubit and nanodrop for purity. Libraries were prepared using the Nextera XT DNA library prep kit (Illumina, San Diego, USA). Sequencing was performed using the Illumina MiSeq system.
Data source location	Research Laboratory for Infectious Diseases, NIHRD, Ministry of Health, Jakarta, Indonesia
Data accessibility	Repository name: DNA Data Bank of Japan Data identification number (permanent identifier): PRJDB12216 Direct link to dataset: https://ddbj.nig.ac.jp/resource/bioproject/PRJDB12216
Related research article	Sunarno, Khariri, F. Muna, K. sariadji, Y. Rukminiati, D. Febriyana, T. Febrianti, R.D.Saraswati, I. Susanti, N. Puspandari, A. Karuniawati, A. Malik, A. Soebandrio. New Approach for the Identification of Potentially Toxigenic *Corynebacterium* sp. Using A Multiplex PCR Assay, J Microb Meth. 184 (2021) 106198. https://doi.org/10.1016/j.mimet.2021.106198

## Value of the Data

• The whole-genome sequencing data of *Corynebacterium diphtheriae* isolated from Indonesia, including strains with sequence types that may originate from Indonesia provide insight on genetic diversity of *Corynebacterium diphtheriae*.
• This data can be analyzed by researchers to understand the molecular epidemiology of this pathogen, especially the molecular typing of some *Corynebacterium diphtheriae* isolated from Indonesia.
• These data provide DNA sequences of *Corynebacterium diphtheriae* as reference sequences to develop and verify molecular methods and can be used for further analyses, such as bacterial virulence factors and antimicrobial resistance.

## 1 Data Description

*Corynebacterium diphtheriae* is a causative agent of diphtheria, an acute infectious disease that usually attacks the upper respiratory system. Diphtheria is characterized by the formation of a distinctive pseudomembrane around the tonsils with several complications, including respiratory obstruction, myocarditis, and neuropathy [1]. The main virulence factor of *C. diphtheriae* is diphtheria toxin, an exotoxin that is responsible for the clinical manifestation and mortality of diphtheria. This toxin is encoded by the *tox* gene and regulated by the *dtxR* gene. The *tox* gene is carried by certain bacteriophages that are inserted into the bacterial chromosome by lysogenesis; therefore, the *tox* gene is only present in the toxigenic type (capable of producing diphtheria toxin) of *C. diphtheriae.* Meanwhile, the *dtxR* gene is found in *C. diphtheriae,* which can be both toxigenic and nontoxigenic [2]*.* Occasionally, there are some ‘anomaly’ types, known as *nontoxigenic tox gene bearing* (NTTB) types. In the NTTB type, the *tox* gene is present, but diphtheria toxin is not synthesized phenotypically and is grouped as a nontoxigenic type [3].

The *tox* and *dtxR* genes are commonly used in laboratory tests for diphtheria using PCR assays. We sought to develop PCR assays with the *tox* and *dtxR* genes as targets for species identification and toxigenicity, including predicting 2 types of NTTB, resulting in an improved method [4]. Here, we present the whole-genome sequencing (WGS) data of 18 *C. diphtheriae* isolates from Indonesia (Table 1). The isolates were collected since 2012 until 2015, mostly have mitis subtype (61%). We used these data to identify SNPs associated with the *tox* and *dtxR* genes to verify the accuracy of the PCR assay [4]. We also used these data for molecular typing using the MLST approach [5]. All isolates were tested positive in Elek test and PCR tox gene, except ind_28 isolate which is the Sequence Type still not determined yet.

**Table 1:** Characterization and identification of 18 *C. diphtheriae* isolates from Indonesia.

No	Sample ID	Isolated Year	Subtype	Elek test	PCR *tox* gene	Sequence Type [5]
1	ind_02	2014	mitis	positive	positive	ST535
2	ind_08	2014	mitis	positive	positive	ST535
3	ind_24	2014	gravis	positive	positive	ST534
4	ind_25	2015	mitis	positive	positive	ST534
5	ind_26	2014	mitis	positive	positive	ST535
6	ind_27	2015	mitis	positive	positive	ST535
7	ind_28	2014	gravis	negative	negative	ND
8	ind_34	2015	mitis	positive	positive	ST534
9	ind_35	2012	mitis	positive	positive	ST377*
10	ind_37	2013	mitis	positive	positive	ST377
11	ind_42	2015	mitis	positive	positive	ST302
12	ind_43	2015	intermedius	positive	positive	ST377
13	ind_44	2015	intermedius	positive	positive	ST377
14	ind_45	2015	mitis	positive	positive	ST534
15	ind_46	2015	mitis	positive	positive	ST534
16	ind_47	2015	intermedius	positive	positive	ST377
17	ind_48	2015	intermedius	positive	positive	ST534
18	ind_49	2015	gravis	positive	positive	ST534

ND=not determined

⁎ The data have not been published

WGS data (FASTQ format) of 18 *C. diphtheriae* isolates have been deposited on DNA Data Bank of Japan (DDBJ) with data identification number: PRJDB12216 (https://ddbj.nig.ac.jp/resource/bioproject/PRJDB12216). These data could be used for further analysis regarding antimicrobial resistance and bacterial virulence factors.

## 2 Experimental Design, Materials and Methods

### 2.1 Isolate Collection and DNA Extraction

Eighteen *C. diphtheriae* were isolated from diphtheria outbreaks in Indonesia from 2012 to 2015 (Table 1). These isolates were randomly selected from Prof. Dr. Sri Oemijati Research Laboratory for Infectious Diseases, Jakarta as one of national reference laboratories. The isolates were obtained from clinical sample of diphtheria cases and their close contacts in some provinces of Indonesia. The archived *C. diphtheriae* isolates were storaged by using TSB + 20% glycerol preservation medium in the ultra-low temperature freezer (-70 to -80 °C). The isolates were revived on blood agar plates and incubated at 37°C overnight. Bacterial species, biotype, and toxigenicity identification were performed by API Coryne (bioMérieux, La Balme les Grottes, France) and Elek tests according to WHO guidelines [6]. One full loop of bacterial colonies was dissolved in 500 μL of Ultrapure DNase/RNase-Free distilled Water (Invitrogen, Waltham, MA, USA). DNA isolation was conducted using a QIAamp DNA Mini Kit (Qiagen, Hilden, Germany) according to the manufacturer's protocol. In the last step, the DNA sample was stored in 50 µl of Ultrapure DNase/RNase-Free distilled Water (Invitrogen, Waltham, MA, USA). The DNA purity was measured using NanoDrop based on the 260/280 nm absorbance value with a ratio of 1.8–2.0. Quantification of DNA was conducted using a Qubit®3.0 Fluorometer (Thermo Fisher Scientific, Waltham, MA, USA) and dsDNA HS Assay Kit (Thermo Fisher Scientific, Waltham, MA, USA). DNA extraction was repeated when the quality or quantity of DNA did not meet the Illumina MiSeq platform requirements.

### 2.2 DNA Library and Whole-Genome Sequencing

DNA libraries were prepared using the Nextera XT DNA Library Prep Kit 2 × 150 bp (Illumina, San Diego, USA) according to the manufacturer's protocol. WGS was conducted using the following steps of the Illumina MiSeq platform: denaturing the libraries; diluting the libraries; preparing the optional PhiX control; loading the libraries onto the reagent cartridge; checking library preparation before inserting into the catridge by KAPA library Quantification Kit Illumina Platform and setting up the sequencing run. The *C. diphtheriae* PW8 complete genome (CP003216.1) was used as a reference sequence.

### 2.3 Data Analysis

Molecular typing was performed with the MLST approach (Table 1). The profiling of 7 loci was performed, and sequence type determination was conducted online via the MLST global database (https://pubmlst.org/). Since 2022, the database was available on https://bigsdb.pasteur.fr/diphtheria/.

## Ethics Statements

The data obtained from archive isolates were exempted from ethical approval as stated by the Health Research Ethics Committee, National Institute of Health Research and Development (HREC-NIHRD): LB.02.01/2/KE216/2017.

## CRediT authorship contribution statement

**Vivi Setiawaty:** Writing – review & editing. **Nelly Puspandari:** Data curation. **Ratih Dian Saraswati:** Visualization. **Dwi Febriyana:** Formal analysis. **Tati Febrianti:** Data curation. **Yuni Rukminiati:** Formal analysis. **Fauzul Muna:** Formal analysis, Data curation. **Fitriana Fitriana:** Writing – review & editing. **Dodi Safari:** Writing – review & editing, Data curation. **Rahadian Pratama:** Writing – review & editing, Data curation. **Lisa Andriani Lienggonegoro:** Writing – review & editing. **Sunarno Sunarno:** Conceptualization, Methodology, Writing – original draft.

## Declaration of Competing Interest

The authors declare that they have no known competing financial interests or personal relationships that could have appeared to influence the work reported in this paper.

## Data Availability

Vaccine-preventable diseases Research (Original data) (DDBJ).

## References

[1] Sharma N.C., Efstratiou A., Mokrousov I., Mutreja A., Das B., Ramamurthy T. (2019). Diphteria. Nat. Rev..

[2] Holmes R.K. (2000). Biology and molecular epidemiology of diphtheria toxin and the tox gene. J. Infect. Dis..

[3] Zakikhany K., Neal S., Efstratiou A. (2014). Emergence and molecular characterisation of non-toxigenic tox gene-bearing *Corynebacteriumdiphtheriae* biovar mitis in the United Kingdom, 2003–2012. Eurosurveillance.

[4] Sunarno K., Muna F., sariadji K., Rukminiati Y., Febriyana D., Febrianti T., Saraswati R.D., Susanti I., Puspandari N., Karuniawati A., Malik A., Soebandrio A. (2021). New approach for the identification of potentially toxigenic *Corynebacterium* sp. using a multiplex PCR assay. J. Microbiol. Methods.

[5] Sunarno S., Rukminiati Y., Saraswati R.D. (2020). ST534: The new sequence type of *Corynebacterium diphtheriae* causing diphtheria in Jakarta and surrounding areas, Indonesia. Turk. J. Med. Sci..

[6] Efstratiou A., Maple C. (1994).

